# Treatment failure and associated risk factors for periprosthetic-joint infections caused by streptococci versus other etiologies: a single-center retrospective cohort study

**DOI:** 10.1186/s42836-025-00341-y

**Published:** 2025-11-24

**Authors:** Arnaud Fischbacher, Jonathan Tschopp, Sylvain Steinmetz, Noémie Boillat-Blanco, Olivier Borens

**Affiliations:** 1 Orthopaedics and Traumatology Service, Lausanne University Hospital (CHUV), 1011 Lausanne, Switzerland; 2 Infectious Diseases Service, Lausanne University Hospital (CHUV), 1011 Lausanne, Switzerland; 3 Bone and Motion, Clinique Bois-Cerf, Hirslanden Private Clinics, 1006 Lausanne, Switzerland

**Keywords:** Periprosthetic-joint infections, Streptococci, Treatment failure

## Abstract

**Background:**

There is no consensus on the antibiotic course or type of surgical treatment in streptococcal periprosthetic-joint infections (PJIs). We aimed to compare the treatment failure rate at 2 years of PJIs caused by streptococci to PJIs caused by other pathogens and identify factors associated with failure.

**Methods:**

It was a single-center retrospective cohort study conducted between 2009 and 2019. We included all patients aged ≥ 18 years undergoing treatment for hip or knee PJI with a 2-year follow-up. We analyzed the treatment failure rate at 2 years of streptococcal PJIs versus PJIs caused by other pathogens with failure defined as a non-successful treatment using the Delphi-based international multidisciplinary consensus of success. We also analyzed factors associated with failure including streptococcal etiology and type of treatment.

**Results:**

We included 404 patients; 62 (15%) had a streptococcal PJI, of which 14 (23%) exhibited treatment failure at 2 years. The treatment failure rate was similar to that for PJIs caused by other pathogens (21%, 71/342) (*P* = 0.78). Streptococci were not associated with failure (OR = 1.55, 95% CI 0.62–3.89, *P* = 0.35). However, *Streptococcus dysgalactiae* (OR = 9.45, 95% CI 1.37–65.46, *P* = 0.02) and debridement, antibiotics and implant retention (DAIR) (OR = 9.31, 95% CI 1.80–48.20, *P* = 0.008) were associated with failure among patients with a streptococcal PJI.

**Conclusions:**

The treatment failure rate of streptococcal PJIs was similar to that for PJIs caused by other pathogens. However, *Streptococcus dysgalactiae* and DAIR were factors associated with failure among patients with a streptococcal PJI. Our results suggest that streptococcal PJIs, especially *Streptococcus dysgalactiae* PJIs, should be surgically treated more aggressively with an implant exchange.

## Introduction

Total hip and knee arthroplasties are expected to increase significantly in the next decades to improve the quality of life of an ever-aging population [1]. Periprosthetic-joint infection (PJI) is one of the most feared complications after arthroplasty, with an incidence of 1% to 2% after primary arthroplasty and 4% to 8% after revision arthroplasty [2, 3]. This complication is associated with prolonged hospital stays, high costs and increased morbidity and mortality [4–8]. Streptococci are responsible for approximately 10% of PJIs and are the second most common bacterial species after staphylococci [9]. Hematogenous spread from a distant focus of infection represents the predominant route of infection. Streptococci were thought to be easy to treat due to their high sensitivity to antibiotics and their acute presentation, making debridement, antibiotics and implant retention (DAIR) possible. However, multiple studies have linked streptococci to an increased risk of recurrence compared to other bacteria with high relapse rates (20–42%), suggesting that these infections may be labeled as “difficult to treat” [9–11]. Some authors even recommend that these infections should be treated with a prolonged course of suppressive antibiotics [12]. The reason for this is still unclear and could be due to the lack of an effective anti-biofilm treatment, knowing that the use of rifampin did not show evidence to improve the outcome [10, 12, 13]. Finally, there is no consensus on the influence of surgical treatment selection with recent studies suggesting that two-stage revision is the treatment of choice [14, 15].

This retrospective study aimed to compare the treatment failure rate of streptococcal PJIs to PJIs caused by other pathogens and identify factors associated with failure.

## Materials and methods

### Study design, setting and participants

We performed a single-center retrospective cohort study in a university hospital in Switzerland between 2009 and 2019. We included all consecutive patients aged ≥ 18 years undergoing treatment for hip or knee PJI (both primary and revision arthroplasties). Patients were identified from the intervention register of the hospital. All included patients had a follow-up medical visit after the second postoperative year and a signed general consent form for their data to be used retrospectively. We excluded patients with unknown 2-year outcome due to loss of follow-up and those who did not give consent for their data to be used.

### Study procedures

We extracted demographics, type of prosthesis, characteristics of the infection, type of surgical intervention and antimicrobial treatment from institutional medical records. Microbiological results including blood cultures, synovial fluid and tissue sample cultures and PCRs (eubacterial PCR 16S) were also extracted. Information on follow-up at 3, 6, 12 and 24 months was extracted from the same institutional medical records.

### Definitions

PJIs were retrospectively defined using the 2021 European Bone and Joint Infection Society (EBJIS) criteria [16]. The infection was classified according to the duration of symptoms at diagnosis as acute (≤ 3 weeks of symptoms) or chronic (> 3 weeks of symptoms). PJIs were furthermore classified into early (< 3 months), delayed (> 3 months to ≤ 2 years) and late (> 2 years) infections according to the time to onset following the implantation or revision surgery [3]. Treatment failure at 2-year follow-up was defined as a non-successful treatment using the Delphi-based international multidisciplinary consensus of success and could be either infection relapse, new infection or death [17]. Infection relapse was defined as the persistence or reappearance of signs and/or symptoms of infection (pain, swelling, redness or wound fistula) during the first two years after surgery. New infection was defined the same way but needed the identification of a different pathogen from the primary PJI.

### Surgical treatment

**Fig. 1:**
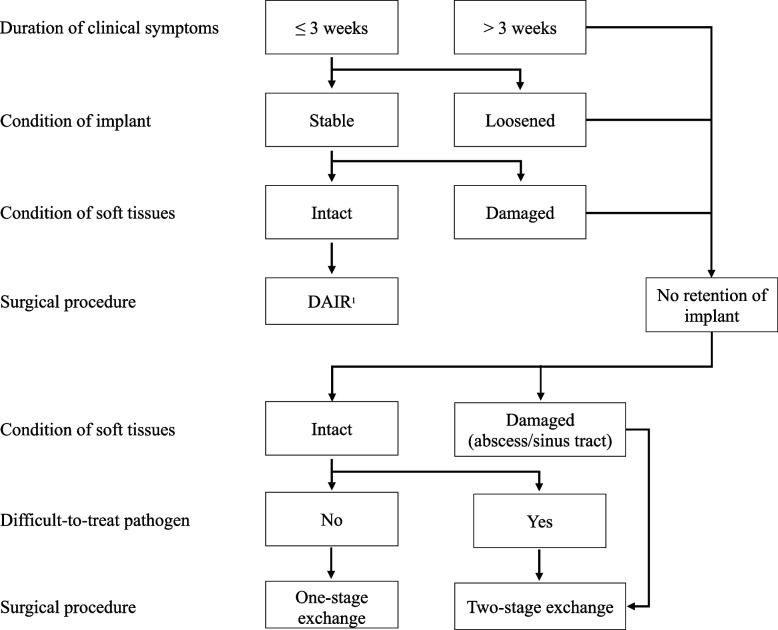
Surgical treatment algorithm (adapted from [3]). ^1^DAIR: debridement, antibiotics, and implant retention

An established treatment algorithm was usually followed after multidisciplinary discussion to decide on the type of surgical treatment (Fig. 1) [3]. Patients with acute infections usually underwent DAIR, with polyethylene exchange systematically performed. Patients with chronic infections, loosened implants or damaged soft tissues usually underwent one- or two-stage exchange depending on the soft tissues (abscess or sinus tract). In case of a two-stage exchange, a longer interval (≥ 6 weeks) was used before reimplantation when a difficult-to-treat pathogen (i.e., with no anti-biofilm therapy available) was documented. Implant removal, joint arthrodesis, or amputation were performed in severely immunocompromised patients, in those with active intravenous drug use, and in cases where arthroplasty would not provide any benefit. Inappropriate surgical treatment was defined as DAIR for a chronic infection.

### Antimicrobial treatment

The medical team usually followed international guidelines on antibiotic treatment [18]. Empiric intravenous antibiotics were started at time of surgery. A switch to targeted antibiotics was usually performed as soon as possible upon reception of microbiological results (tissue samples and sonication) and susceptibility testing. The intravenous treatment was given for 1–2 weeks depending on the clinical evolution and current guidelines and was followed by an oral regimen for a total of 12 weeks. In case of a two-stage exchange, antibiotics were continued until re-implantation without an antibiotic-free interval. A prolonged course of suppressive antibiotics was never used. The type of antibiotic treatment was recorded as well as the use of anti-biofilm treatment (i.e., at least one day of rifampin for Gram-positive cocci and fluoroquinolones for Gram-negative bacilli). Streptococcal PJIs were treated with rifampin until published data showed no evidence of benefit in the outcome [10, 12, 13].

### Data analyses

Statistical analyses were performed with STATA 16® (Statacorp, Texas). Continuous variables are presented as median with interquartile range (IQR) and categorical variables as numbers with percentage. We compared demographics, type of prosthesis, characteristics of the infection and type of intervention and antimicrobial treatment between patients with streptococcal PJIs and those with PJIs caused by other pathogens using Mann Whitney-U, *chi*-squared, or Fisher exact tests, as appropriate. A *P*-value ≤ 0.05 was considered statistically significant. To identify factors associated with treatment failure in the whole study population and in patients with streptococcal PJIs, we used a multivariate logistic regression model with backward selection and a significance level of *P* ≥ 0.10 for removal from the model.

## Results

### Study population

Of 456 consecutive screened patients with PJI, 37 (8%) were excluded due to lack of consent and 15 (3%) due to loss of follow-up within 2 years after PJI. Five (10%) of these 52 excluded patients had streptococcal PJIs. A total of 404 patients were included in the study; 62 (15%) had streptococcal PJIs (Table 1). Among patients with streptococcal PJIs, 56% had hip PJIs and 44% knee PJIs. Patients with streptococcal PJIs were older at time of diagnosis and more comorbid. Streptococcal PJIs were more often acute than non-streptococcal PJIs.

**Table 1:** Demographic and clinical data of streptococcal versus non-streptococcal PJIs (*n* = 404 patients)

	**Streptococcal PJIs** ***N* = 62 (15%)**		**Non-streptococcal PJIs** ***N* = 342 (85%)**		***P***
Demographics^1^					
Age, median (IQR)	75	(66–81)	69	(61–77)	**0.004**
Males, *n* (%)	31	(50)	189	(55)	0.44
Charlson Comorbidity Index, median (IQR)	4	(3–6)	4	(2–5)	**0.03**
BMI, median (IQR)	29	(24–33)	29	(25–33)	0.58
Type of prosthesis, *n* (%)					0.70
Hip	35	(56)	202	(59)	
Knee	27	(44)	140	(41)	
Type of infection, *n* (%)					**0.03**
Acute	36	(58)	148	(43)	
Chronic	26	(42)	194	(57)	
Infection timing, *n* (%)					0.26
Early	13	(21)	73	(21)	
Delayed	34	(55)	153	(45)	
Late	15	(24)	116	(34)	
Type of intervention, *n* (%)					0.33
DAIR	22	(35)	121	(35)	
One-stage exchange	3	(5)	43	(13)	
Two-stage exchange	34	(55)	152	(44)	
Implant removal	3	(5)	22	(6)	
Arthrodesis	0		2	(1)	
Amputation	0		2	(1)	
Use of anti-biofilm antibiotics^2^, *n* (%)	8	(13)	239	(70)	** < 0.001**
Treatment failure after 2 years, *n* (%)	14	(23)	71	(21)	0.78
Infection relapse (%)	9	(64)	36	(51)	
New infection (%)	3	(21)	19	(27)	
Death (%)	2	(14)	16	(22)	

IQR: interquartile range, BMI: body mass index, DAIR: debridement, antibiotics, and implant retention

^1^Race or ethnicity information was not available; ^2^At least one day of either rifampin or fluoroquinolones

### Microbiology

Among the 404 patients, 353 (87%) had a documented pathogen. 62 (15%) had a streptococcal monomicrobial PJI. For non-streptococcal PJIs, the most isolated bacteria were coagulase-negative staphylococci followed by *Staphylococcus aureus* and Gram-negative bacilli. Of note, three polymicrobial infections included streptococci. The detailed distribution of bacterial species is provided in Table 2.

**Table 2:** Microbiological findings of PJIs (*n* = 404 patients)

**Streptococcal species**	***N*** **= 62**	**(15%)**
*Streptococcus pyogenes*	4	(6%)
*Streptococcus agalactiae*	17	(28%)
*Streptococcus dysgalactiae*	16	(26%)
*Streptococcus* bovis group	2	(3%)
*Streptococcus* viridans group	23	(37%)
**Other bacteria species**	***N* = 342**	**(85%)**
*Staphylococcus aureus*^1^	88	(26%)
Coagulase-negative staphylococci	107	(31%)
Enterococci	12	(4%)
Other Gram-positive cocci^2^	2	(1%)
Gram-negative bacilli^3^	36	(11%)
Anaerobes^4^	21	(6%)
Fungi	1	(1%)
Polymicrobial infections^5^	24	(7%)
Culture-negative infections	51	(15%)

^1^80 methicillin-sensitive *S. aureus* (MSSA), 8 methicillin-resistant *S. aureus* (MRSA)

^2^1 *Bacillus cereus*, 1 *Corynebacterium striatum*

^3^14 *E. coli*, 3 *Citrobacter koseri*, 3 *Proteus mirabilis*, 1 *Proteus vulgaris*, 5 *Pseudomonas aeruginosa*, 2 *Enterobacter cloacae*, 3 *Klebsiella aerogenes*, 3 *Klebsiella pneumoniae*, 3 *Campylobacter fetus*

^4^14 *Cutibacterium acnes*, 1 *Propionibacterium avidum*, 1 *Granulicatella*, 1 *Bacterioides fragilis*, 1 *Bacterioides vulgatus*, 1 *Fingoldia magna*, 1 *Clostridium celerecrescens*, 1 *Ruminococcus gnavus*

^5^1 *Streptococcus dysgalactiae*, 1 *Streptococcus agalactiae*, 1 *Streptococcus* viridans group

### Treatment

Table 1 summarizes the surgical and antimicrobial treatment. In streptococcal PJIs, surgical treatment consisted of DAIR in 22 (35%) patients (with polyethylene exchange systematically performed), one-stage exchange in 3 (5%), two-stage exchange in 34 (55%), and implant removal in 3 (5%). The only treatment difference between streptococcal and non-streptococcal PJIs was that streptococcal PJIs were treated less often with anti-biofilm antibiotics (13% vs. 70%, *P* < 0.001). Only two streptococcal PJIs were treated with DAIR and anti-biofilm antibiotics, with treatment failure in both.

### Treatment failure at 2 years of streptococcal PJIs versus PJIs caused by other pathogens

Among the 62 streptococcal PJIs, treatment failure at 2-year follow-up was observed in 14 (23%) patients. Infection relapse occurred in 9 (64%) patients and new infection in 3 (22%). Two patients (14%) died. The treatment failure rate was similar to that for PJIs caused by other pathogens (21%, 71/342) (*P* = 0.78) (Table 1). We also found a similar failure rate between streptococcal PJIs and PJIs caused by other pathogens after one-stage exchange (0/3, 0% vs. 6/43, 14%, *P* = 0.49), two-stage exchange (3/34, 9% vs. 24/152, 16%, *P* = 0.30), and DAIR (9/22, 41% vs. 34/121, 28%, *P* = 0.35).

### Factors associated with treatment failure at 2 years

Streptococci infection was not associated with treatment failure in univariate (OR = 0.88, 95% CI 0.41–1.89, *P* = 0.73) or multivariate logistic regression analyses (OR = 1.55, 95% CI 0.62–3.89, *P* = 0.35) (Table 3). Only polymicrobial infections were associated with treatment failure in univariate (OR = 3, 95% CI 1.18–7.64, *P* = 0.02) and multivariate analyses (OR = 6.71, 95% CI 2.14–21.06, *P* = 0.001). Conversely, risk of treatment failure was reduced in culture-negative infections in univariate (OR = 0.06, 95% CI 0.21–0.54, *P* = 0.001) and multivariate analyses (OR = 0.07, 95% CI 0.01–0.58, *P* = 0.01).

Regarding the surgical treatment, DAIR was associated with treatment failure in univariate (OR = 2.87, 95% CI 1.76–4.69, *P* = 0.001) and multivariate analyses (OR = 11.27, 95% CI 3.58–35.50, *P* = 0.001). Risk of treatment failure was reduced with the use of anti-biofilm antibiotics in univariate analysis (OR = 0.61, 95% CI 0.38–0.99, *P* = 0.05) but not in the multivariate model.

**Table 3:** Factors associated with 2-year treatment failure: univariate and multivariate analyses

	**Cure** ***N* = 319 (79%)**		**Failure** ***N* = 85 (21%)**		**Univariate analysis**			**Multivariate analysis**		
					**OR**	**95% CI**	*P*	**OR**	**95% CI**	*P*
Demographics										
Age, median (IQR)	70	(62–78)	71	(62–80)	1.01	0.99–1.03	0.38			
Males, *n* (%)	169	(53)	51	(60)	1.33	0.82–2.17	0.25			
Charlson, Comorbidity Index, median (IQR)	4	(2–5)	4	(2–5)	1.01	0.89–1.14	0.93			
BMI, median (IQR)	29	(25–33)	29	(26–33)	1.02	0.98–1.06	0.28			
Type of prosthesis, *n* (%)										
Hip	185	(58)	52	(61)		reference				
Knee	134	(42)	33	(39)	0.88	0.53–1.43	0.60			
Type of infection, *n* (%)										
Acute	135	(42)	49	(58)		reference			reference	
Chronic	184	(58)	36	(42)	0.54	0.33–0.87	**0.012**	3.20	1.02–10.10	0.05
Infection timing, *n* (%)										
Early	67	(21)	19	(22)		reference				
Delayed	156	(49)	31	(37)	0.70	0.37–1.33	0.28			
Late	96	(30)	35	(41)	1.29	0.68–2.44	0.44			
Use of antibiofilm antibiotics^1^, *n* (%)	203	(64)	44	(52)	0.61	0.38–0.99	0.05			
Type of intervention, *n* (%)										
Non-DAIR^2^	223	(70)	38	(45)		reference				
DAIR	96	(30)	47	(55)	2.87	1.76–4.69	** < 0.001**	11.27	3.58–35.50	** < 0.001**
Inappropriate surgery	4	(1)	3	(4)	2.88	1.31–13.13	0.17			
Microbiology										
* Staphylococcus aureus*^3^	66	(21)	22	(26)		reference			reference	
Coagulase-negative staphylococci	88	(28)	19	(22)	0.65	0.32–1.29	0.22	1.38	0.58–3.29	0.47
* Streptococcus* spp	48	(15)	14	(16)	0.88	0.41–1.89	0.73	1.55	0.62–3.89	0.35
* Enterococcus* spp	9	(3)	3	(4)	1.00	0.25–4.03	1.00	1.51	0.27–8.35	0.64
Other Gram positive cocci^4^	2		0							
Gram-negative bacilli^5^	27	(8)	9	(11)	1.00	0.41–2.45	1.00	1.33	0.47–3.77	0.59
Anaerobes^6^	17	(5)	4	(5)	0.71	0.21–2.32	0.57	1.33	0.35–5.06	0.67
Fungi	0		1							
Polymicrobial infections^7^	12	(4)	12	(14)	3	1.18–7.64	**0.02**	6.71	2.14–21.06	**0.001**
Culture-negative infections	50	(16)	1	(1)	0.06	0.21–0.54	** < 0.001**	0.07	0.01–0.58	**0.01**

IQR: interquartile range, BMI: body mass index, DAIR: debridement, antibiotics, and implant retention

^1^At least one day of either rifampin or fluoroquinolones

^2^ Cure: one-stage exchange (*n* = 40), two-stage exchange (*n* = 159), implant removal (*n* = 20), arthrodesis (*n* = 2), amputation (*n* = 2)/Failure: one-stage exchange (*n* = 6), two-stage exchange (*n* = 27), implant removal (*n* = 5), arthrodesis (*n* = 0), amputation (*n* = 0)

^3^80 methicillin-sensitive *S. aureus* (MSSA), 8 methicillin-resistant *S. aureus* (MRSA)

^4^1 *Bacillus cereus*, 1 *Corynebacterium striatum*

^5^14 *E. coli*, 3 *Citrobacter koseri*, 3 *Proteus mirabilis*, 1 *Proteus vulgaris*, 5 *Pseudomonas aeruginosa*, 2 *Enterobacter cloacae*, 3 *Klebsiella aerogenes*, 3 *Klebsiella pneumoniae*, 3 *Campylobacter fetus*

^6^14 *Cutibacterium acnes*, 1 *Propionibacterium avidum*, 1 *Granulicatella*, 1 *Bacterioides fragilis*, 1 *Bacterioides vulgatus*, 1 *Fingoldia magna*, 1 *Clostridium celerecrescens*, 1 *Ruminococcus gnavus*

^7^1 *Streptococcus dysgalactiae*, 1 *Streptococcus agalactiae*, 1 *Streptococcus* viridans group

### Factors associated with treatment failure at 2 years among patients with streptococcal PJIs

*S. dysgalactiae* infection was associated with treatment failure (6 relapses and 2 new infections in 16 patients, 50%) in univariate (OR = 6.67, 95% CI 1.40–31.72, *P* = 0.02) and multivariate analyses (OR = 9.45, 95% CI 1.37–65.46, *P* = 0.02) (Table 4). The frequency of treatment failure was higher in patients with a streptococcal PJI treated with DAIR compared to other surgical treatments (9/22, 41% vs. 5/40, 12%) making DAIR a factor associated with failure in univariate (OR = 4.85, 95% CI 1.37–17.17, *P* = 0.01) and multivariate analyses (OR = 9.31, 95% CI 1.80–48.20, *P* = 0.008). Risk of treatment failure was not reduced with the use of anti-biofilm antibiotics (OR = 2, 95% CI 0.50–8.00, *P* = 0.33). Finally, late presumably hematogenous infections were not associated with treatment failure (OR = 0.80, 95% CI 0.17–3.77, *P* = 0.78).

**Table 4:** Factors associated with 2-year treatment failure among patients with a streptococcal PJI

	**Cure** ***N* = 48 (77%)**		**Failure** ***N* = 14 (23%)**		**Univariate analysis**			**Multivariate analysis**		
					**OR**	**95% CI**	*P*	**OR**	**95% CI**	*P*
Demographics										
Age, median (IQR)	75	(67*–*81)	73	(62*–*81)	0.98	0.93*–*1.03	0.44			
Males, *n* (%)	21	(44)	10	(71)	3.21	0.88*–*11.70	0.07			
Charlson, Comorbidity Index, median (IQR)	5	(4*–*6)	4	(2*–*7)	0.98	0.74*–*1.30	0.90			
BMI, median (IQR)	29	(24*–*34)	30	(26*–*32)	1.03	0.93*–*1.14	0.54			
Type of prosthesis, *n* (%)										
Hip	27	(56)	8	(57)		reference				
Knee	21	(44)	6	(43)	0.96	0.29*–*3.21	0.95			
Type of infection, *n* (%)										
Acute	25	(52)	11	(79)		reference				
Chronic	23	(48)	3	(21)	0.30	0.07*–*1.20	0.09			
Infection timing, *n* (%)										
Early	8	(17)	5	(36)		reference				
Delayed	30	(62)	4	(28)	0.21	0.05*–*0.98	0.05			
Late	10	(21)	5	(36)	0.80	0.17*–*3.77	0.78			
Use of antibiofilm antibiotics^1^, *n* (%)	8	(17)	4	(29)	2	0.50*–*8.00	0.33			
Inappropriate surgery	1	(2)	0							
*Streptococcus* species, *n* (%)										
* Streptococcus* viridans group	20	(42)	3	(21)		reference			reference	
* Streptococcus pyogenes*	4	(8)	0							
* Streptococcus agalactiae*	14	(29)	3	(21)	1.43	0.24*–*8.14	0.69	1.34	0.15*–*12.25	0.79
* Streptococcus dysgalactiae*	8	(17)	8	(57)	6.67	1.40*–*31.72	**0.02**	9.45	1.37*–*65.46	**0.02**
* Streptococcus* bovis group	2	(4)	0							
Type of intervention, number (%)										
Non-DAIR^2^	35	(73)	5	(36)		reference			reference	
DAIR	13	(27)	9	(64)	4.85	1.37*–*17.17	**0.01**	9.31	1.80*–*48.20	**0.008**

IQR: interquartile range, BMI: body mass index, DAIR: debridement, antibiotics, and implant retention

^1^At least one day of either rifampin or fluoroquinolones

^2^Streptococcal PJIs: one-stage exchange *n* = 3, two-stage exchange *n* = 34, implant removal *n* = 3/Non-streptococcal PJIs: one-stage exchange *n* = 43, two-stage exchange *n* = 152, implant removal *n* = 22, arthrodesis *n* = 2, amputation *n* = 2

## Discussion

In this study, the 2-year treatment failure rate was similar for streptococcal PJIs and non-streptococcal PJIs (23% vs. 21%, *P* = 0.78). Only polymicrobial infections were associated with treatment failure, and culture-negative infections with a reduced risk of failure, as already demonstrated [19, 20]. This result does not corroborate previous studies, which showed a higher failure rate in streptococcal PJIs [9–12, 14, 15]. However, our comparable failure rate with other etiologies could be explained by a greater proportion of two-stage exchanges for streptococcal PJIs in our cohort. While 58% of these infections were classified as acute, surgical treatment consisted of DAIR in only 35%. The surgical treatment algorithm was therefore not always followed, and streptococcal PJIs were treated more aggressively over time based on the expertise of the medical team. Recent studies showed a worse prognosis than previously published for streptococcal PJIs managed by DAIR [14, 15, 21]. The time from the onset of symptoms to debridement and performing polyethylene exchange might be a confounding factor. Nevertheless, these results are in line with our study in which DAIR was associated with failure, although polyethylene exchange was systematically performed and DAIR was considered inappropriate in only one patient. This finding supports that DAIR should be avoided in streptococcal PJIs when the microbiology is available before surgery.

Regarding microbiology, we identified a streptococcal species that was associated with a higher failure rate: *Streptococcus dysgalactiae*. This is consistent with a recent study, which found that *Streptococcus dysgalactiae* was the most common organism isolated in their failure group [14]. Two previous studies showed that *Streptococcus agalactiae* was also associated with a higher failure rate, but it was not the case in our study [9, 15]. The role of anti-biofilm antibiotics against streptococcal biofilms is still controversial. Several studies demonstrated that streptococci were able to produce biofilm [22–24]. However, it has been shown that the addition of rifampin does not improve the outcome, which is in accordance with our findings [10, 12–15]. Further research is needed on microbiological differences of streptococci species, such as biofilm formation, as *Streptococcus dysgalactiae* seems to behave differently. Lack of effective biofilm-active antibiotics may explain the higher failure rate in *Streptococcus dysgalactiae* PJIs. *Streptococcus dysgalactiae* could thus be considered a “difficult to treat” pathogen and should be surgically treated more aggressively with an implant exchange. A prolonged course of suppressive antibiotics, as recommended by some authors, could also be used when *Streptococcus dysgalactiae* is found after DAIR [12].

Finally, we acknowledge the limitations of our study. The study’s reliance on medical records prevented us from recording the length of the antimicrobial treatment. The results may have been distorted if the antibiotic therapy was interrupted, particularly anti-biofilm treatment. It also prevented us from identifying hematogenous infections. We presumed that late streptococcal PJIs were hematogenous. They were not associated with treatment failure, contrary to previous studies, probably due to a lack of power [14]. The accuracy of the PJIs’ definition based on criteria introduced after the collection of data might be questioned, but to overcome this limitation, all patients’ files were reviewed by an infectious disease specialist. Our findings are also limited due to the small number of cases for some streptococcal species and the observational nature of the study, with no randomization of the surgical treatment. However, some strengths of the study should be highlighted, such as the considerable number of included patients and the homogeneity regarding the surgical and antimicrobial treatment throughout the study period.

## Conclusions

The treatment failure rate of streptococcal PJIs was similar to that for PJIs caused by other pathogens. However, *Streptococcus dysgalactiae* and DAIR were factors associated with failure among patients with a streptococcal PJI. Our results suggest that streptococcal PJIs, especially *Streptococcus dysgalactiae* PJIs, should be surgically treated more aggressively with an implant exchange.

## Data Availability

All data generated and analyzed during this study are included in this published article (tables) and are available from the corresponding author upon reasonable request.
